# Resonant Gas Sensing in the Terahertz Spectral Range Using Two-Wire Phase-Shifted Waveguide Bragg Gratings

**DOI:** 10.3390/s23208527

**Published:** 2023-10-17

**Authors:** Yang Cao, Kathirvel Nallappan, Guofu Xu, Maksim Skorobogatiy

**Affiliations:** 1 Center for Advanced Laser Technology, Hebei University of Technology, 5340 Xiping Road, Tianjin 300401, China; 2 Engineering Physics, Polytechnique Montréal, C.P. 6079, Succ. Centre-Ville, Montréal, QC H3C 3A7, Canada; kathirvel.nallappan@polymtl.ca (K.N.); guofu.xu@polymtl.ca (G.X.)

**Keywords:** terahertz technology, gas sensing, plasmonic waveguide, phase-shifted grating, additive manufacturing

## Abstract

The development of low-cost sensing devices with high compactness, flexibility, and robustness is of significance for practical applications of optical gas sensing. In this work, we propose a waveguide-based resonant gas sensor operating in the terahertz frequency band. It features micro-encapsulated two-wire plasmonic waveguides and a phase-shifted waveguide Bragg grating (WBG). The modular semi-sealed structure ensures the controllable and efficient interaction between terahertz radiation and gaseous analytes of small quantities. WBG built by superimposing periodical features on one wire shows high reflection and a low transmission coefficient within the grating stopband. Phase-shifted grating is developed by inserting a Fabry–Perot cavity in the form of a straight waveguide section inside the uniform gratings. Its spectral response is optimized for sensing by tailoring the cavity length and the number of grating periods. Gas sensor operating around 140 GHz, featuring a sensitivity of 144 GHz/RIU to the variation in the gas refractive index, with resolution of 7 × 10^−5^ RIU, is developed. In proof-of-concept experiments, gas sensing was demonstrated by monitoring the real-time spectral response of the phase-shifted grating to glycerol vapor flowing through its sealed cavity. We believe that the phase-shifted grating-based terahertz resonant gas sensor can open new opportunities in the monitoring of gaseous analytes.

## 1. Introduction

An increasing demand for the monitoring of air quality has promoted the development of high-performance gas sensing devices operating on various chemical and physical principles such as optical, calorimetric, chromatographic, acoustic, as well as electrochemical [1,2,3,4,5]. Among those, optical sensors exhibit unique advantages by being immune to electromagnetic interferences, free of external power supply, capable of operating in harsh environments, and allowing multiplexed remote sensing [6,7,8]. Furthermore, for various gaseous analytes (e.g., gases, vapors, aerosols), the terahertz band is abundant with spectral fingerprints [9,10,11,12], thus opening new opportunities in optical gas sensing. As a complementary technique to the well-established infrared spectroscopy that probes electronic transitions in molecules [13], THz spectroscopy rather probes molecular vibrations, which are particularly pronounced in the gas phase [14]. Additionally, to handle the submillimeter radiation, THz optics are usually much larger than infrared ones, thus enabling novel designs (e.g., integrate with gas cell) and fabrication techniques (e.g., additive manufacturing) of gas sensing devices. However, a significant challenge for gas sensing, particularly at low analyte concentrations, is the weak signal, which prompts the use of long straight gas cells [15,16] or circular multi-pass cells [17,18] to obtain the measurable absorption, thus resulting in large and cumbersome gas sensor systems.

It is, therefore, important to investigate the integrated resonant structures, particularly in the THz band, capable of reducing the size of sensor systems, compared to the free-space systems, without sacrificing sensitivity. One way to achieve this is by using hollow core waveguides filled with gaseous analytes to perform broadband molecular vibration absorption spectroscopy. Such waveguides operate using various guidance principles (e.g., ARROW, bandgap, plasmonic) and offer high field–analyte overlap [19,20,21,22] while occupying much smaller volumes (e.g., coiled hollow core fibers [23,24]) than free-space gas cells. They are predominantly used to monitor the frequency-dependent imaginary part (loss) of the analyte Refractive Index (RI). Therefore, for chemical species identification and component differentiation, one usually resorts to the costly THz optical sources supporting stable and broadband operation.

Alternatively, a THz waveguide-based sensor of relatively short length can be designed using various resonant elements in their structures (e.g., Bragg gratings, asymmetric directional couples, integrated Fabry–Perot resonant cavities, and coherent scattering elements [25,26,27,28,29,30,31]). Due to the low bandwidth nature of resonant devices, one can then monitor the gaseous analyte RI (mostly its real part) by tracking the spectral position of various singularities using cost-effective THz sources (e.g., resonant tunneling diodes).

Although high sensitivities are readily achievable by both one-dimensional (e.g., photonic crystal cavity on silicon wafer [26]) and two-dimensional resonators (e.g., pillar arrays [29]), it is noted that for most reported optical sensors, the gaseous analyte delivery infrastructure comes as an afterthought. In contrast, in this work, this crucial component is co-engineered with optical ones, thus ensuring the independent efficient operation of both with minimal mutual intrusion for gas sensing. This subtly integrated structure outperforms the conventional open-structured sensors in terms of compactness and performance stability. Particularly, by removing the employment of external gas cells, the proposed sensor is especially suitable for monitoring small quantities of gaseous analytes.

In this work, we propose a real-time resonant THz gas sensor based on phase-shifted waveguide Bragg grating (WBG). At the core of this device is a broadband two-wire plasmonic waveguide formed by metalizing polymer cylinders that are encapsulated within a closed polymer cage. The gaseous analyte flows inside the cage and in the air gap of a two-wire plasmonic waveguide. WBG is formed by a periodic conical pattern imprinted onto one of the cylinders of a two-wire waveguide and is optimized to feature a spectrally broad stopband. Finally, the phase-shifted grating is formed by inserting a Fabry–Perot cavity in the form of a uniform waveguide section in the middle of WBG. The cavity length and the number of grating periods should be chosen to support a single spectrally narrow transmission peak within a broad WBG stopband. The THz spectral response of phase-shifted gratings is then studied for different lengths of a cavity and different refractive indices of gaseous analyte that are filling the semi-sealed cavity. By tracking the position of the transmission peak, our sensor sensitivity near 0.14 THz is found to be ~14.5 GHz/mm for changes in the cavity length, and ~144 GHz/RIU for changes in the analyte RI (real part). A theoretical sensing resolution of ~7 × 10^−5^ RIU is estimated from the 10 MHz resolution of our spectrometer. Finally, using a continuous-wave (CW) THz spectroscopy system, we experimentally demonstrate the real-time detection of glycerol vapors from an electronic cigarette as an analyte. Namely, when replacing dry air with glycerol vapor in the cavity of a phase-shifted grating module, a shift in the sensor resonant frequency (transmission peak) of ~50 MHz reveals an RI difference of ~3.5 × 10^−4^ RIU.

Different from the most reported optical gas sensors whose delicate structures are realized using costly infrastructures (e.g., femtosecond laser and deep reactive ion etchers), the proposed gas sensor on a centimeter-scale THz waveguide can be rapidly manufactured using the emerging 3D printing technology with precision and robustness. Owing to the ubiquitous availability of hardware as well as the compact modular design that integrates various crucial elements, we believe that this sensor confronts a lower threshold for entering into production and less challenging engineering problems for operation in practical applications.

## 2. Two-Wire Waveguide Bragg Gratings

**Figure 1:**
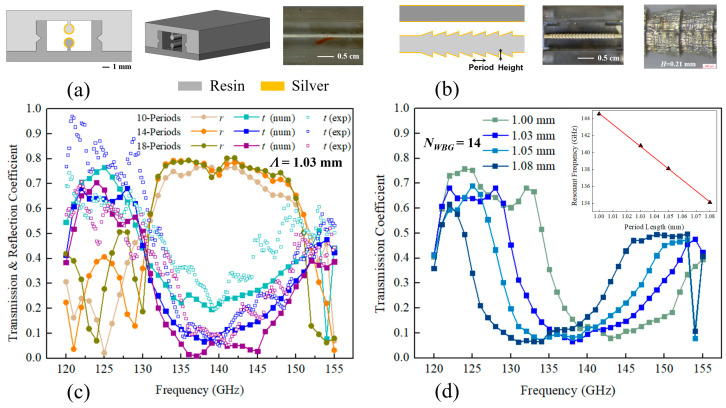
Micro-encapsulated two-wire waveguide and WBG fabricated using stereolithography and wet chemistry deposition. (**a**) Schematic of an encapsulated two-wire waveguide. (**b**) The two-wire WBG features a sequence of end-to-end connected truncated cones written on one of the two wires. (**c**) Transmission and reflection spectra of WBGs featuring a different number of periods, *Λ* = 1.03 mm. (**d**) Numerical transmission spectra of WBGs for different period lengths, *N_WBG_* = 14. Inset: The center frequency of a WBG stopband as a function of its period length.

Unlike the conventional two-wire metallic waveguides [32,33], the two-wire waveguides used in this work and detailed in [34] feature a modular design with the wires in the form of metalized polymer cylinders encapsulated within a polymer enclosure (see Figure 1a). Such a micro-encapsulated design circumvents the intrinsic engineering defect of conventional one in alignment, and promises mechanical stable, cost-effective, and highly reconfigurable THz optical circuits for various applications (the comparison of transmission spectra is shown in Figure 2d in [34]). The waveguide cross-sectional design, including the wire diameter, the air gap size, as well as the topography of the enclosure, were carefully tailored to ensure the featureless transmission spectra with low insertion loss for a several-centimeter-long waveguide around 140 GHz. Such a design eliminates the presence of spectral ripples and enables distinct measured transmission spectra using THz spectroscopy, thus facilitating the signal identification for gas sensing.

Additionally, the integration of the plasmonic terahertz waveguide and semi-sealed cavity promises the controllable interaction between the supported THz surface plasmon polariton wave and the gaseous analyte flowing through. However, as refractive indices (real part) for most gases are close to one, it is challenging to detect the difference between them, thus necessitating the use of long interaction distances (long gas cells) to accumulate sufficient phase differential between different analytes. In contrast, by using resonant devices like a Fabry–Perot cavity (in this work: realized in the form of a phase-shifted WBG), we can fold the optical path to realize much smaller devices.

Experimentally, we find that the two-wire WBGs featuring a sequence of end-to-end connected truncated cones on one wire was an optimal design that can be printed reliably with high precision and without supports, using a tabletop stereolithography 3D printer (see Appendix A for details in fabrication). In principle, one can further increase the grating strength (stopband bandwidth) by using other geometries such as deep rectangular grooves on both wires. However, it is noted that realizing such designs is challenging due to microstructure deformation induced by the intrinsic cure-through defect of 3D printing and the difficulty of aligning such structures [35].

Specifically, the UV radiation in each exposure not only cures the resin within the top printed layer, but also leaks through the cured layer and solidifies some resin on the other side. Therefore, the resultant cumulative deformation has to be taken into consideration for the grating structure design, as it becomes explicit for prints where geometry changes rapidly from one layer to another. Additionally, the two-wire waveguide components were manually assembled from two complementary 3D-printed parts. When subwavelength features are superimposed on both parts, the postprocessing facet-polishing step can easily lead to their misalignments in practice. Furthermore, the optimal truncated ridge height was found to be ~0.2 mm, enabling a large bandwidth of the stopband, manageable loss in the passband, as well as the reproducible optical performance of printed WBGs (see Figure 1b).

For a stopband center frequency of ~140 GHz, the period of WBGs is found to be *Λ* = 1.03 mm. The transmission and reflection spectra for the 2.5 cm long WBGs containing *N_WBG_* = 10, 14, 18 periods are shown in Figure 1c, with numerical transmission and reflection coefficients in the vicinity of the stopband center frequency reaching <0.1 and >0.75 values, respectively, when the number of periods is over 14. The linear dependence of the stopband center frequency on the grating period *Λ* is shown in Figure 1d for a 14-period structure, with a slope of 131 GHz/mm. Experimentally, the transmission measurements were conducted using a CW-THz spectroscopy system (see Appendix A for details in characterization), and the spectral response of the 3D printed THz WBGs within the grating stopband agrees well with numerical simulation, as seen in Figure 1c. A minimal transmission coefficient of ~0.08 was found for the ~16 GHz wide grating stopband of a 14-period WBG.

**Figure 2:**
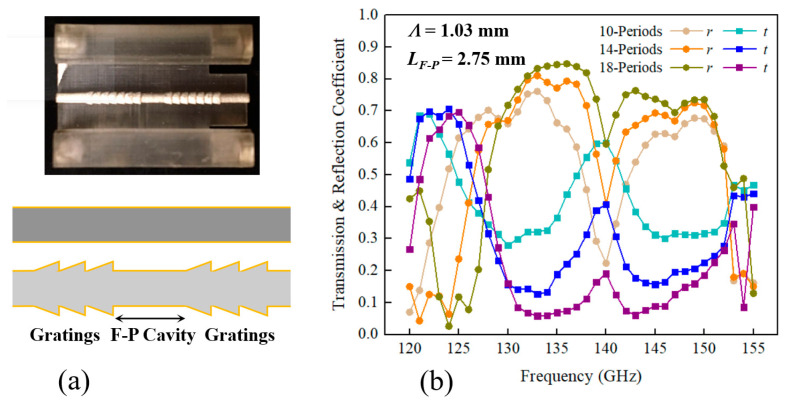
Phase-shifted waveguide Bragg grating. (**a**) Schematic and photo of a two-wire waveguide-based phase-shifted WBG. (**b**) Numerical transmission and reflection spectra of a phase-shifted WBG as a function of the number of periods, *Λ* = 1.03 mm and *L_F-P_* = 2.75 mm.

**Figure 3:**
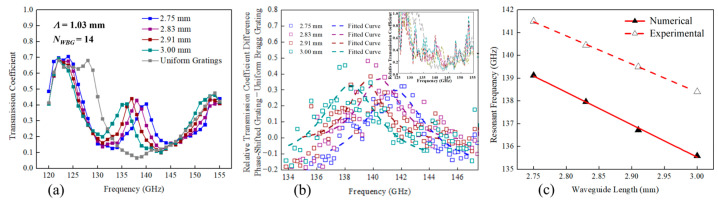
Spectral response of phase-shifted WBGs for various cavity lengths with *N_WBG_* = 14 and *Λ* = 1.03 mm. (**a**) Numerical transmission spectra and (**b**) experimental normalized transmission spectra of phase-shifted WBGs. Inset: the transmission spectra of phase-shifted WBGs and a uniform WBG. (**c**) The spectral position of the transmission peak within the WBG stopband as a function of the cavity length.

Next, we realize a narrow transmission window within the WBG stopband by incorporating a Fabry–Perot cavity, which is a two-wire waveguide section with a length of *L_F-P_* = 2.75 mm, between two WBG reflectors. The resonance in the Fabry–Perot cavity results in the presence of transmission peaks within the WBG stopband. Experimentally, we find that a 14-period phase-shifted WBG shown in Figure 2a results in a superior performance in terms of the transmission spectra for gas sensing. It is worth noting that the elongation of the grating leads to a narrower transmission peak (~2 GHz bandwidth for a phase-shifted WBG containing 18 periods), but comes at the cost of deteriorated transmission peak intensity (~0.1 transmission coefficient difference between the resonant frequency and other frequencies within the grating stopband), thus posing challenges in identifying the desired transmission peak. Additionally, in a numerical simulation, the bandwidth of the exclusive transmission peak decreases from ~4.7 GHz to ~3.6 GHz when the waveguide length increases from ~0.5*Λ* to ~2.5*Λ*. Further reduction in bandwidth by extending the waveguide section is infeasible due to the appearance of multiple spectral singularities within the grating stopband, while the spectral position of the transmission peak with a basically unaffected bandwidth moves toward a lower frequency when the F-P cavity length slightly increases (see Figure 3a).

Because of the standing waves formed inside of the photomixer silicon lenses and free-space cavities of the CW-THz spectroscopy setup, parasite ripples are superimposed on measured transmission spectra [36], posing challenges in identifying the transmission peak of phase-shifted WBGs from the experimental data. To simplify the task, we identify the resonant peak position by subtracting the transmission spectrum of a uniform WBG from the spectra of the phase-shifted WBGs (see Figure 3b). A good correspondence between experiment and theory is found for the spectral position of a transmission peak as a function of the cavity length, with an exception of a small systematic frequency shift of ~2 GHz as seen in Figure 3c. We believe that this consistent discrepancy is mainly attributed to the structural nonuniformity of experimental gratings, which results in the longer equivalent F-P cavity compared with that of the ideal numerical model. Both in theory and experiment, the dependence is linear, with a slope of ~14.5 GHz/mm.

## 3. Two-Wire Waveguide-Based Resonant Gas Sensor

**Figure 4:**
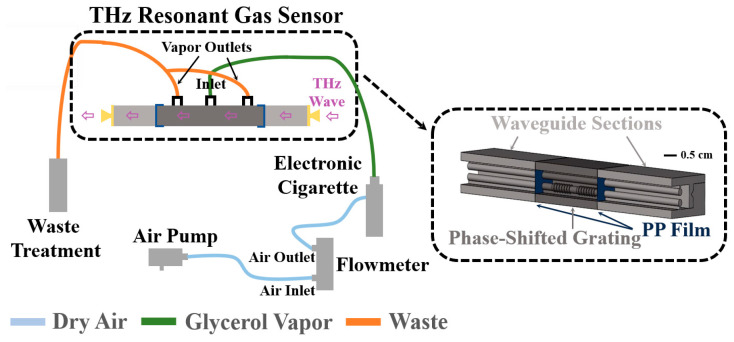
Schematic of the experimental setup to fill the cavity hosting two metalized wires with glycerol vapor.

Finally, we demonstrate real-time THz gas sensing based on our thus-designed phase-shifted WBG. A 2.5 cm long phase-shifted grating module containing a cavity of *L_F-P_* = 2.75 mm in the middle of 14-period gratings with *Λ* = 1.03 mm was sealed on both ends with polyethylene film (*α* < 0.01 cm^−1^ for a lower-terahertz band) with a thickness of tens of micrometers. In experiments, the addition of such a THz transparent material led to negligible changes in the transmission spectra of this module. To couple with the free-space THz beam for characterization, this module was placed between two 3 cm long featureless two-wire waveguide sections which support broadband operation. The assembled waveguide component was then fitted with conical horn antenna and placed inside the THz spectroscopy setup (see Figure 4). It is noted that three through holes were drilled on the side wall of the enclosure of the phase-shifted grating module for gaseous analyte delivery.

Glycerol is one of the main ingredients of vaping liquid, to which nicotine and flavors are added. The gas mixture generated by electronic cigarettes is notoriously harmful to human health. Specifically, glycerol aerosol alone has been shown to have an impact on the liver and energy metabolism [37]. Therefore, detecting glycerol vapor in air is of practical significance in health management, which was demonstrated by the proposed sensor in this work. In experiments, glycerol vapor generated by an electronic cigarette was introduced into the 0.6 mL volume flow cell through the inlet in the middle of a cell with a constant flow rate of ~20 mL/s, while the waste vapor was removed from the two ends of a flow cell through the outlets for waste treatment. The well-designed location of inlet and outlet openings as well as the short voiding time allow the cavity to completely replace its filled gas in sub seconds, enabling the real-time monitoring of gas RI changes.

**Figure 5:**
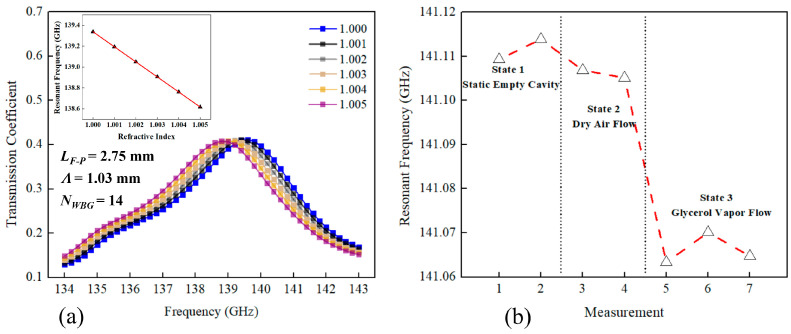
The spectral response of a phase-shifted WBG with gaseous analytes of different RIs in the cavity. (**a**) Numerical transmission spectrum of a phase-shifted WBG in the vicinity of a resonant peak for different values of gaseous analyte RI. Inset: The spectral position of the transmission peak as a function of the analyte RI. A slope of ~144 GHz/RIU can be found in the linear fit (red line). (**b**) Experimental time dependence of the spectral position of the transmission peak. Its variation can be found in the red dotted line.

The numerical simulations of the independent phase-shifted grating module predict that the spectral position of a transmission peak is linear with the gaseous analyte RI with the corresponding sensitivity of 144 GHz/RIU (see Figure 5a). Given the 10 MHz resolution of our CW-THz spectrometer, the theoretical resolution of our sensor is then estimated to be 7 × 10^−5^ RIU, which is as much as an order of magnitude lower than the RI difference between most common gases (e.g., the difference is on the level of 10^−3^ to 10^−4^ RIU) [38]. In experiments, the transmission spectra of a phase-shifted WBG with an empty cavity, the cavity with dry airflow, and the cavity with glycerol vapor flow were measured subsequently. In dynamic measurements covering the spectral range of a transmission peak, the scanning time for a single data point was ~10 s to alleviate the impact of the inherent latency of a CW spectroscopy system using lock-in acquisition, and to ensure fine spectra with 10 MHz resolution. The center position of a transmission peak was found by first fitting a data cloud of the normalized phase-shifted WBG transmission spectra within the grating stopband using smooth Lorentzian lineshapes,

(1) $$T_{N\mathrm{orm}}(\upsilon ,\upsilon_{center},\Delta \upsilon ,A,T_{0})=T_{0}+A\frac{\Delta \upsilon }{4{\left(\upsilon -\upsilon_{center}\right)}^{2}+\Delta \upsilon^{2}}$$

and then finding the spectral position of the fit maximum *υ_center_*, similarly to what is shown in Figure 3b.

A typical sensor readout is presented in Figure 5b from which we see that the spectral position of the transmission peak is relatively stable in continuously recorded transmission spectra of the same analyte. Additionally, for an empty cell or a cell with a flow of dry air, the position of the transmission maximum also remains practically unchanged, indicating the immunity of the proposed sensor to changes in gas flow rate. At the same time, when introducing the glycerol vapor, the transmission peak shifts by ~50 MHz, which corresponds to the RI change of ~3.5 × 10^−4^ compared to that of dry air. Highly consistent experimental results were obtained in each measurement of this sensor. Owing to its compact integrated structure and insensitivity to the environment change, this two-wire waveguide-based sensor can find its practical applications in gas sensing by simply replacing the external infrastructure for gas delivery (see the setup out of the black dotted region in Figure 4). For instance, one can detect the concentration of explosive or toxic gas flowing in pipelines or dispersed in the air remotely in petrochemical industry.

## 4. Discussion

In this work, we propose micro-encapsulated two-wire plasmonic waveguide-based phase-shifted Bragg gratings and demonstrate their applications in real-time THz gas sensing. End-to-end connected truncated cones with a ridge height of ~0.2 mm superposed on one of the two wires were chosen as an optimal WBG design. Low transmission and high reflection coefficients were found within the ~16 GHz wide stopband of such WBGs. Phase-shifted WBG featuring a Fabry–Perot cavity was then developed by placing a uniform waveguide section in the center of a WBG. A single narrow transmission peak of ~3.6 GHz (HFWM) bandwidth in the middle of a WBG stopband was realized by using a ~2.75 mm long cavity flanked on both sides by two seven-period WBGs with a Q-factor of ~39. The theoretical sensitivity of the peak spectral position to changes in the RI of gaseous analytes inside the 2.5 cm long phase-shifted WBG is estimated to be 144 GHz/RIU. The response of our sensor to glycerol vapor flow at low concentrations was then verified in a proof-of-concept time-resolved experiment, which reliably detected the displacement of dry air by glycerol vapor with a resultant RI change of ~3.5 × 10^−4^ RIU.

For future work, we note that higher sensitivity sensor designs are readily achievable by moving the sensor operational frequency to higher frequencies [39], while also increasing the number of periods in the WBG to reduce the spectral width of a transmission peak. The long-term stability of the proposed sensor also needs to be characterized and further optimized for practical applications. Additionally, considering the modular and reconfigurable design of micro-encapsulated two-wire waveguide components, the sensing of selectivity is readily available via collaboration with THz waveguide-based spectroscopy [19] for various monitoring applications of gaseous analytes such as trace gas analysis and detection [40].

## Data Availability

Data associated with this research are available and can be obtained from authors upon reasonable request.

## Appendix A

The proposed micro-encapsulated two-wire plasmonic waveguide components were split into two complementary parts, which exhibit one metalized wire attached to a half dielectric cage. We fabricated the dielectric support of such designed structures using an SLA 3D printer (Asiga Freeform PRO2) with the waveguide direction corresponding to its Z-axis. To suppress deformations associated with the cure-through-resin effect, the parts having periodical subwavelength features superimposed on wires were printed along the single direction from the cone smaller base toward its larger base using the finest layer thickness (10 μm). After 3D printing, we protected the cage’s inner surface and then deposited a silver layer onto the uncovered wire support through wet chemistry deposition. Finally, two selectively metalized parts were assembled into a two-wire waveguide component by matching the V-shaped groove and ridge on each cage.

The proposed THz two-wire waveguide-based components, including uniform and phase-shifted WBGs, were characterized using a free-space CW-THz spectroscopy system (Toptica Photonics TeraScan 1550). Tunable THz radiation corresponding to the beat frequency between two C-band distributed feedback lasers was generated in the emitter photomixer. Transmitted through waveguide components, the amplitude of the THz signal was recorded by the receiver photomixer using lock-in detection. In experiments, WBG modules were placed between two two-wire waveguide sections fitted with WR6.5 conical horn antennas (Virginia Diodes). Their transmission coefficients were calculated by comparing the measured transmission spectra of this assembly with the reference corresponding to a two-wire waveguide assembly of the same length.
